# Small Peptide Derived from SFRP5 Suppresses Melanogenesis by Inhibiting Wnt Activity

**DOI:** 10.3390/cimb46060324

**Published:** 2024-05-29

**Authors:** Yoon-Seo Choi, Jin-Gwen Hong, Dong-Young Lim, Min-Seo Kim, Sang-Hoon Park, Hee-Cheol Kang, Won-Sang Seo, Jongsung Lee

**Affiliations:** 1 Graduate School-Interdisciplinary Program in Biocosmetics, Sungkyunkwan University, Suwon 16419, Republic of Korea; eveelf@g.skku.edu; 2 Research and Development Department, Benex Co., Ltd., Cheongju 28118, Republic of Korea; benex-royal@naver.com; 3 R&D Center, Supadelixir Co., Ltd., Chuncheon 24232, Republic of Korea; limdy83@naver.com (D.-Y.L.); ms.kim@supadelixir.com (M.-S.K.); 4 Department of Plastic Surgery, ID Hospital, Gangnam 06039, Republic of Korea; spark@idhospital.com; 5 Materials Division Affiliated Research Center, GFC Life Science Co., Ltd., Hwaseong 18471, Republic of Korea; michael@gfcos.co.kr; 6 Molecular Dermatology Laboratory, Department of Integrative Biotechnology, College of Biotechnology and Bioengineering, Sungkyunkwan University, Suwon 16419, Republic of Korea

**Keywords:** Sfrp5pepD, Wnt, MITF, β-catenin, pigmentary disorders

## Abstract

Melanocytes, located in the epidermis’ basal layer, are responsible for melanin pigment production, crucial for skin coloration and protection against UV radiation-induced damage. Melanin synthesis is intricately regulated by various factors, including the Wnt signaling pathway, particularly mediated by the microphthalmia-associated transcription factor (MITF). While MITF is recognized as a key regulator of pigmentation, its regulation by the Wnt pathway remains poorly understood. This study investigates the role of Sfrp5pepD, a peptide antagonist of the Wnt signaling pathway, in modulating melanogenesis and its potential therapeutic implications for pigmentary disorders. To tackle this issue, we investigated smaller peptides frequently utilized in cosmetics or pharmaceuticals. Nevertheless, there is a significant scarcity of reports on peptides associated with melanin-related signal modulation or inhibiting melanin production. Results indicate that Sfrp5pepD effectively inhibits Wnt signaling by disrupting the interaction between Axin-1 and β-catenin, thus impeding downstream melanogenic processes. Additionally, Sfrp5pepD suppresses the interaction between MITF and β-catenin, inhibiting their nuclear translocation and downregulating melanogenic enzyme expression, ultimately reducing melanin production. These inhibitory effects are validated in cell culture models suggesting potential clinical applications for hyperpigmentation disorders. Overall, this study elucidates the intricate interplay between Wnt signaling and melanogenesis, highlighting Sfrp5pepD as a promising therapeutic agent for pigmentary disorders. Sfrp5pepD, with a molecular weight of less than 500 Da, is anticipated to penetrate the skin unlike SFRPs. This suggests a strong potential for their use as cosmetics or transdermal absorption agents. Additional investigation into its mechanisms and clinical significance is necessary to enhance its effectiveness in addressing melanin-related skin conditions.

## 1. Introduction

Melanin is essential for the formation of coat color in mammals. The coloration of mammalian fur, eyes, and hair primarily depends on the type, amount, quality, and distribution of melanin [1]. Melanocytes, located in the basal layer of the epidermis, produce and transfer melanin pigments to keratinocytes, affecting skin color. Melanin in keratinocytes protects against UV radiation, reducing the risk of DNA damage, skin cancer, and premature aging [2]. Melanin synthesis takes place in melanosomes within pigment cells. The process is regulated by enzymes including tyrosinase (TYR), tyrosinase-related protein 1 (TRP-1), and dopachrome tautomerase (DCT) (or tyrosinase-related protein 2 (TRP-2)), which convert tyrosine into melanin pigments [3,4]. Tyrosinase is essential in melanin production, as it catalyzes the initial and rate-limiting step of melanogenesis [5].

Melanogenesis undergoes intricate regulation influenced by a multitude of intrinsic and extrinsic factors originating from the environment or neighboring skin cells. These factors encompass UV radiation, melanocyte-stimulating hormone (MSH) [6,7], agouti signal protein (ASP), endothelin 1 (ET1), as well as a diverse range of growth factors and cytokines [8,9]. It is well-established that various signaling pathways play critical roles in controlling the regulatory mechanisms of different molecules and elucidating the relationship between phenotypes. For instance, melanogenesis is intricately linked to specific signaling pathways, including -MSH/MC1R, Wnt/β-catenin, SCF/c-KIT, and ubiquitin activating enzyme (ET-1)/ETB-R [10]. Within the Wnt/β-catenin signaling pathway, Wnt1 and Wnt3a are key regulators of microphthalmia-associated transcription factor (MITF) expression and its downstream genes, TYR and TRP-1, thereby influencing melanogenesis [11,12]. β-catenin enhances the transcriptional expression of MITF by directly interacting with LEF1, thereby promoting the proliferation and differentiation of melanoblasts and exerting a significant impact on melanogenesis [13].

Wnt plays various roles in neural crest formation, influencing induction, migration, proliferation, and differentiation [14]. Mice deficient in Wnt-1 and Wnt-3 lack pigment cells, potentially due to improper expansion of early neural crest cells [15]. Recent discoveries have revealed a physical interaction between CREB and β-catenin upon PKA/cAMP pathway activation in normal human melanocytes and B16-F0 mouse melanoma cells, resulting in functional cooperation on the MITF promoter [16]. Furthermore, β-catenin not only participates in Lef1-dependent control of MITF gene transcription but also interacts with the MITF protein [17].

The main factor responsible for regulating tyrosinase and tyrosinase-related proteins is MITF [18]. MITF expression is triggered by the activation of the melanocyte differentiation inducer such as MSH [19]. MITF, recognized as the master regulator of pigmentation, is a target of the Wnt pathway [20]. The Wnt family of secreted glycoproteins activates different intercellular signaling cascades through its receptor, frizzled, including the canonical Wnt/β-catenin pathway and the noncanonical Wnt pathways [21].

However, the effects of Wnt/β-catenin pathway modulation on adult melanogenesis and pigmentary disorders are not extensively explored. In cell culture, targeted suppression of β-catenin expression leads to reduced levels of melanocyte differentiation-associated markers. Elevated levels of Dickkopf-1 (DKK1), an inhibitor of the canonical Wnt signaling pathway, contribute to low pigmentation levels in specific areas of human adults by suppressing β-catenin and MITF [22].

Secreted frizzled-related proteins (SFRPs) interact with Wnt ligands, regulating canonical and noncanonical pathways [23]. Sfrps inhibit the Wnt/β-catenin pathway and demonstrate distinct characteristics. Understanding the interplay between MITF- and Wnt-associated pathways in melanogenesis remains limited.

SFRPs are the largest family of Wnt inhibitors. The founding member Frzb was initially identified through its sequence similarity with the Fz receptors [24,25], and immediately associated with Wnt signaling because of its ability to bind to Wnt8 and block its signaling in Xenopus, strongly supporting its role as a Wnt antagonist [25,26]. Concurrently, additional members of the family were isolated either through sequence homology with Fz receptors [27] or independently of Wnt activity. Belonging to the family of secreted proteins [28], secreted frizzled-related protein 5 (SFRP5) shares structural similarities with the Fz receptor in the Wnt signaling pathway. By competitively inhibiting the Fz receptor, SFRP5 effectively suppresses the activity of the Wnt signaling pathway [28], establishing its reputation as a well-known inhibitor of this pathway. SFRP5 is a known antagonist of the Wnt signaling pathway [29]. SFRP5 is known to regulate the development of various tissues and organs, but its role in vitiligo, characterized by skin depigmentation, has been unclear due to its inhibition of Wnt signaling. A recent study suggested that SFRP5 could affect melanogenesis in vitiligo by regulating Wnt signaling [30]. Overexpression of SFRP5 was found in vitiligo skin lesions compared to normal melanocytes. Altering SFRP5 levels in melanocytes showed that its overexpression suppressed melanin synthesis by downregulating MITF and its targets through Wnt/β-catenin signaling inhibition, while silencing SFRP5 increased melanin synthesis and activated Wnt signaling [30].

The use of SFRPs itself as a transdermal absorption agent or cosmetic ingredient is subject to limitations. Generally, SFRPs have a molecular weight ranging between 30 and 40 kDa [31]. However, peptides that are absorbed by the skin need to be 500 Da or less [32]. This means that SFRP5 itself cannot be absorbed through the skin. For this reason, a smaller peptide that retains only the active site of the SFRP5 function is needed.

To address this, we sought answers from smaller peptides commonly used in cosmetics or pharmaceuticals. Bioactive peptides exhibit diverse biological and cosmetic or pharmaceuticals functions, leading to their classification into four main categories: signal peptides, carrier peptides, neurotransmitter-inhibitory peptides, and enzyme-inhibitory peptides, based on their primary characteristics [33]. Among them, the peptides used in cosmetics or the skincare industry mainly belong to the category of signal peptides. One of the pioneering cosmetic signal peptides is the palmitoyl peptide (Pal–Lys–Thr–Thr–Lys–Ser), which demonstrates collagen-modulating abilities for anti-wrinkle and wound healing purposes [34]. Another signal peptide known for stimulating collagen synthesis is palmitoyl tripeptide-5 (palmitoyl–Lys–Val–Lys). It can emulate the action of thrombospondin-1, a naturally occurring molecule that induces the binding of the sequence Lys–Arg–Phe–Lys to the inactive form of transforming growth factor-β (TGF-β), thereby facilitating the release of the active form of TGF-β [35]. However, reports regarding peptides related to melanin-related signal modulation or melanin production inhibition are very scarce.

The aim of this study is to validate the melanin inhibition resulting from SFRP5, especially from the peptide Sfrp5pepD, and to evaluate its potential as a melanin inhibitor or therapeutic agent in the cosmetic or pharmaceutical industries. Consequently, our goal is to investigate the feasibility of developing peptides smaller than SFRP5 that exhibit enhanced skin penetration effectiveness.

## 2. Materials and Methods

### 2.1. Material Information

α-MSH was procured from Sigma (St. Louis, MO, USA). The Wnt agonist was acquired from EMD Millipore (Billerica, MA, USA). The cell counting kit-8 was sourced from Dojindo Molecular Technologies (Kumamoto, Japan). Antibodies against tyrosinase, actin, Trp-1, Trp-2, MITF, phospho-CREB, CREB, phospho-AKT, AKT, and β-catenin were obtained from Santa Cruz Biotechnology (Dallas, TX, USA). Antibodies against phospho-GSK3β and GSK3β substrates were purchased from Cell Signaling Technology (Danvers, MA, USA). Peptides were synthesized using an automatic peptide synthesizer (PeptrEX-R48, Peptron, Daejeon, Republic of Korea) following the manufacturer’s recommended protocol. Purification and analysis of the synthesized polypeptides were conducted using reverse-phase high-performance liquid chromatography (Prominence LC-20AB, Shimadzu, Kyoto, Japan) and mass spectrometer (HP1100 Series LC/MSD, Hewlett-Packard, Roseville, CA, USA). After undergoing a series of peptide selection experiments, Sfrp5pepD was chosen for further testing. Subsequently, it was mass-produced by Benex Co., Ltd. (Cheongju, Republic of Korea), Supadelixir Co., Ltd. (Chuncheon, Republic of Korea), and GFC Life Science Co., Ltd. (Hwaseong, Republic of Korea) under collaborative efforts. The mass-produced product of Sfrp5pepD is the key active ingredient in a product named ActivePep WT200^TM^ (or Winhibin, or Pep-LumiSkin), and its amino acid sequence is recognized as Acetyl–Alanine-Glutamine-Lysine amide (Ac-Ala-Gln-Lys-amide). When calculated based on the molecular weight of the final mass-produced product, a concentration of 10 µM Sfrp5pepD is equivalent to 3.87 ppm (the recommended prescription concentration of Sfrp5pepD in final cosmetic formular is 5–10 ppm.). The International Nomenclature of Cosmetic Ingredients (INCI) name of Sfrp5pepD can be verified through the certificates of Benex Co., Ltd., GFC Life Science Co., Ltd., or Supadelixir Co., Ltd.

### 2.2. Cell Culture

B16 murine melanoma cells, Dulbecco’s Modified Eagle Medium (DMEM), and fetal bovine serum (FBS) were acquired from the American Type Culture Collection (ATCC; Manassas, VA, USA). B16 cells were maintained in DMEM supplemented with 10% FBS and incubated at 37 °C in a 5% CO_2_ atmosphere. Normal human melanocytes (NHMs) sourced from lightly pigmented adult skin donors were obtained from Cascade Biologics (#C-024-5C, Portland, OR, USA) and cultured in Medium 254 (#M-254-500; Cascade Biologics, Portland, OR, USA) supplemented with Human Melanocyte Growth Supplement (HMG) (#S-002-5; Cascade Biologics, Portland, OR, USA). Melanocytes at passage numbers between 4 and 7 were utilized for the experiments.

### 2.3. Proliferation Assay

Cell proliferation was assessed using a CCK-8 based assay. B16F1 melanoma cells were seeded at 5 × 10^3^ per well in 96-well culture plates and incubated in medium containing 10% FBS. After 24 h, cells were treated with α-MSH (0.1 μM) or peptides (0.1, 1, 10 mM) in 90 μL of SFM for 24, 48, and 72 h. At the indicated time points, cells were washed once and incubated with 10 μL of CCK-8 solution at 37 °C for 2 h. Then, the absorbance was measured at 450 nm using a microplate reader (VersaMax; Molecular Devices, San Jose, CA, USA). Independent experiments were repeated in triplicate.

### 2.4. L-DOPA Staining

The L-DOPA reactivity of NHMs was evaluated in situ using cultures fixed with 4% paraformaldehyde in PBS for 40 min at room temperature. Following permeabilization in 0.1% Triton X-100 in PBS for 2 min, cells were rinsed with PBS and then incubated in 0.1% L-DOPA for 3 h at 37 °C. Subsequently, cells were washed with PBS, and images were captured using an Olympus Fluoview FV1000 microscope (Olympus, Tokyo, Japan).

### 2.5. Melanin Content

Cell pellets were solubilized in 10% DMSO, dissolved in 1 N NaOH at 80 °C, and boiled for 2 h. Melanin concentrations were determined by comparing the OD at a wavelength of 490 nm with a standard curve obtained from synthetic melanin. To calculate the actual melanin formation per cell, the total melanin content of each pellet was divided by the number of melanoma cells. All measurements were conducted in triplicate.

### 2.6. Tyrosinase Activity

B16F1 cells were cultured with α-MSH (0.1 μM) for 72 h and then lysed with 150 μL of a lysis buffer containing 0.1 M sodium phosphate (pH 6.8), 1% Triton X-100, and 0.1 mM phenylmethylsulfonyl fluoride (PMSF) for 20 min at 4 °C. The lysates were centrifuged at 12,000× *g* for 30 min, and the supernatant containing equal amounts of proteins (30 μg) was used to measure the rate of oxidation of L-DOPA. After quantifying the protein levels using a BCA protein assay (Pierce, Rockford, IL, USA) and adjusting the concentration with lysis buffer, each lysate was placed in a well of a 96-well plate, and 100 μL of substrate solution (1 mM L-DOPA in PBS, pH 6.8) was added. Following incubation for 2 h at 37 °C, tyrosinase activity was analyzed spectrophotometrically by monitoring the oxidation of DOPA to dopachrome at 450 nm using an ELISA plate reader (VersaMax; Molecular Devices, San Jose, CA, USA).

### 2.7. RNA Extraction and Real-Time PCR

Total RNA was extracted from cell lysates using the Total RNA Purification Kit (Nanohelix, Daejeon, Republic of Korea), and cDNA was synthesized using an RT-premix (Elpisbio, Daejeon, Republic of Korea) according to the manufacturer’s instructions. Oligonucleotide primers were purchased from Bioneer (Daejeon, Republic of Korea). For reverse-transcription-PCR (RT-PCR), the PCR primers’ sequences were as follows: The primer sequences for Mouse Tyrosinase are: Forward 5′-GGCCAGCTTTCAGGCAGAGGT-3′, Reverse 5′-TGGTGCTTCATGGGCAAAATC-3′; for Mouse TRP-1: Forward 5′-GGATGACCGTGAGCAATGGC-3′, Reverse 5′-CGGTTGTGACCAATGGGTGC-3′; for Mouse TRP-2: Forward 5′-CACTCACGGCAAATTCAACGGCAC-3′, Reverse 5′-GACTCCACGACATACTCAGCAC-3′; for Mouse GAPDH: Forward 5′-CACTCACGGCAAATTCAACGGCAC-3′, Reverse 5′-GACTCCACGACATACTCAGCAC-3′; for Human Tyrosinase: Forward 5′-CACATACAGCAAGCCCAA-3′, Reverse 5′-CAGTGCTCTTGCTTCAGA-3′; for Human TRP-1: Forward 5′-AGCAGTAGTTGGCGCTTTGT-3′, Reverse 5′-TCAACCAGGTGGTTTTGTGA-3′; for Human TRP-2: Forward 5′-TGCCACAACATGGATGAACT-3′, Reverse 5′-TTGTGCCCACCAAACAGTAA-3′; and for Human GAPDH: Forward 5′-TGCCACAACATGGATGAACT-3′, Reverse 5′-TTGTGCCCACCAAACAGTAA-3′. PCR products were separated by electrophoresis on 1% agarose gels and visualized under UV light. For quantitative real-time PCR (qRT-PCR), experiments were performed using GENE ROTOR Q. Target gene expression was normalized against the expression of the housekeeping gene, GAPDH. Relative quantification was performed using the comparative ∆∆Ct method according to the manufacturer’s instructions.

### 2.8. Western Blot

Cells were lysed in a lysis buffer (50 mM Tris-HCl, pH 7.4, 150 mM NaCl, 1 mM EDTA, 5 mM sodium orthovanadate, 1% NP-40, and a protease inhibitor cocktail) for 30 min on ice and then centrifuged (13,000 rpm, 20 min, 4 °C). Supernatants were collected, and the total protein concentration was determined using a Bradford assay kit (Bio-Rad, Hercules, CA, USA). Equal amounts of protein were loaded and separated by 10% sodium dodecyl sulfate polyacrylamide gel electrophoresis (SDS-PAGE), transferred to a PVDF membrane, and probed with appropriate antibodies. Primary antibodies were detected by incubating with HRP-conjugated secondary antibodies (1:10,000 in 0.5% bovine serum albumin) using an ECL system.

### 2.9. In Situ Proximity Ligation Assay (PLA)

Cells grown on coverslips were fixed with 4% paraformaldehyde for 15 min, permeabilized with 0.1% Triton X-100 for 5 min, and then blocked with blocking solution (Olink Bioscience, Uppsala, Sweden) for 30 min at 37 °C. Two primary antibodies were incubated with antibody diluent (Olink Bioscience) for 30 min at 37 °C. After washing three times in wash buffer A, cells were incubated with positive and negative PLA probes (Olink Bioscience) for 1 h at 37 °C. After washing, cells were incubated with ligation solution (Olink Bioscience) for 30 min at 37 °C. Cells were washed two times in wash buffer A and then incubated in amplification solution (Olink Bioscience) for 100 min at 37 °C. After washing in wash buffer B, cells were mounted with mounting solution (Olink Bioscience) containing DAPI. Photographs were taken using a confocal microscope (Olympus Fluoview FV1000; Olympus, Tokyo, Japan).

### 2.10. Statistical Analysis

The findings are based on at least four separate experiments and are presented as the mean ± standard error of the mean (SEM). Group comparisons were conducted using ANOVA, followed by Tukey’s HSD post hoc test for statistical significance. Differences were considered statistically significant at * *p* < 0.05.

## 3. Results

### 3.1. The Identification of Wnt Antagonist Peptides

**Figure 1:**
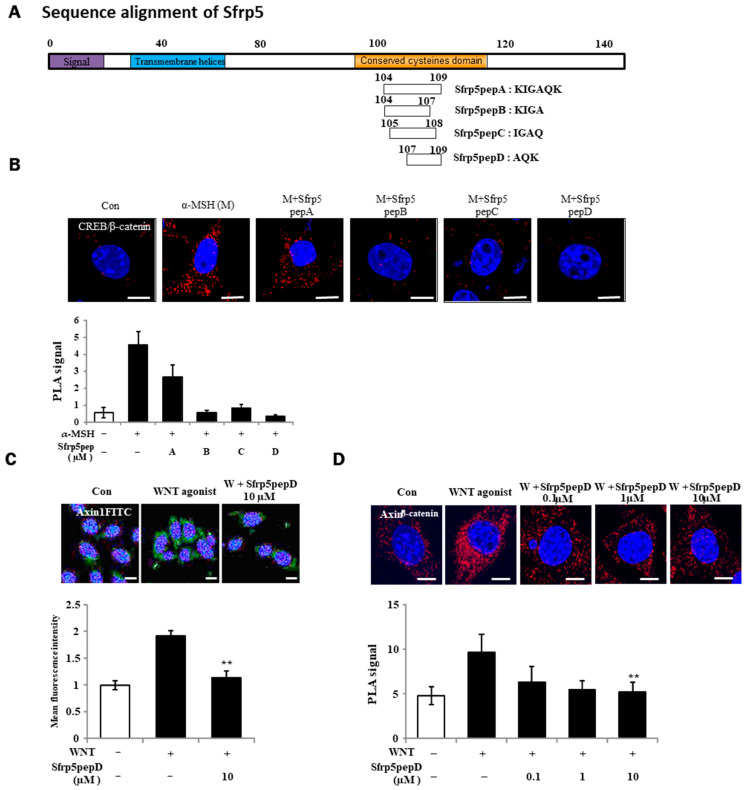
Identification of a Wnt antagonist peptide. (**A**) Illustrative depiction of WNT antagonist peptide variants. (**B**,**D**) B16F1 cells were exposed to α-MSH or Sfrp5 peptides at 1 μM for 12 h. Following fixation, in situ PLA was conducted for CREB/β-catenin and Axin-1/β-catenin using specific antibodies. Nuclei were counterstained with DAPI (blue). Red spots indicate physical interactions between the two specified molecules. (**C**) Immunostaining of β-catenin in B16F1 melanoma cells using a 60× objective. Treatment with Wnt agonist for 3 h stabilized β-catenin protein in the presence or absence of Sfrp5pepD. Significant differences were found when comparing with WNT agonist-treated control: ** *p* < 0.01. Scale bar = 10 μm.

Using a sequence search on the binding domain of the Wnt5 protein, we synthesized four Wnt antagonist peptide samples with molecular weights below 500 Da to assess their agonistic effects on Wnt activation (Figure 1A). Particularly important here is the conserved cysteine domain, which was considered crucial for the characteristics of Wnt antagonists. Four overlapping peptides were generated based on this.

For the identification of Wnt antagonist peptides (A, B, C, D), we investigated the physical interactions of CREB/β-catenin in B16F1 melanoma cells treated with α-MSH or Wnt agonist peptides using a PLA (see Figure 1B). Sfrp5pepD yielded the most prominent result. For this reason, Sfrp5pepD was selected to continue the experiments. Wnt signaling prompts the formation of the β-catenin destruction complex, which includes Axin-1 and GSK-3β [36]. Thus, we initially examined the expression of Axin-1, the pivotal scaffold protein of this complex, through immunofluorescence. Sfrp5pepD effectively decreased the localization induced by the Wnt agonist (see Figure 1C). Subsequently, the impact of Sfrp5pepD on the interaction between Axin-1 and β-catenin was evaluated using in situ PLA analysis. Sfrp5pepD significantly impeded the interaction of Axin-1 and β-catenin in a dose-dependent manner (see Figure 1D). These findings suggest that Sfrp5pepD obstructed Wnt signaling. Since SFRP5 effectively suppresses the activity of the Wnt signaling pathway [28], it can be confirmed that Sfrp5pepD derived from SFRP5 also possesses the same Wnt inhibitory mechanism. Furthermore, this indicates that Sfrp5pepD derived from SFRP5 has secured the region with precise activity. Inhibiting the Wnt signaling pathway leads to reduced melanin production. SFRP5 was overexpressed in the skin lesions of vitiligo patients. Indeed, SFRP5 inhibits melanin synthesis in melanocytes in vitiligo by suppressing the Wnt/β-catenin signaling pathway [30]. Although Sfrp5pepD is a newly created peptide under 500 Da [32], unlike SFRP5, it has not been proven in melanin-related experiments. For this reason, experiments were conducted to confirm the correlation between MITF/β-catenin or tyrosinase and related enzymes involved in melanin production following Wnt.

### 3.2. Sfrp5pepD Suppressed Not Only the Interaction of MITF/β-Catenin but Nuclear Translocation of MITF and β-Catenin

Previous research has demonstrated that Wnt/β-catenin signaling stabilizes the interaction between MITF and β-catenin [37,38,39]. Wnt can indeed regulate MITF at the protein degradation level, irrespective of its transcription [40]. The expression of DKK1, an inhibitor of canonical Wnt/β-catenin signaling, is responsible for inhibiting melanocyte pigmentation by suppressing β-catenin and MITF expression [13]. In the case of SFRP5, the related mechanism has been proven. SFRP5 overexpression inhibited melanin synthesis in PIG1 cells by downregulating MITF [30]. However, this report indicates that increased SFRP5 within the cells affects MITF; it does not report that externally added SFRP5 or related fragments downregulate MITF.

**Figure 2:**
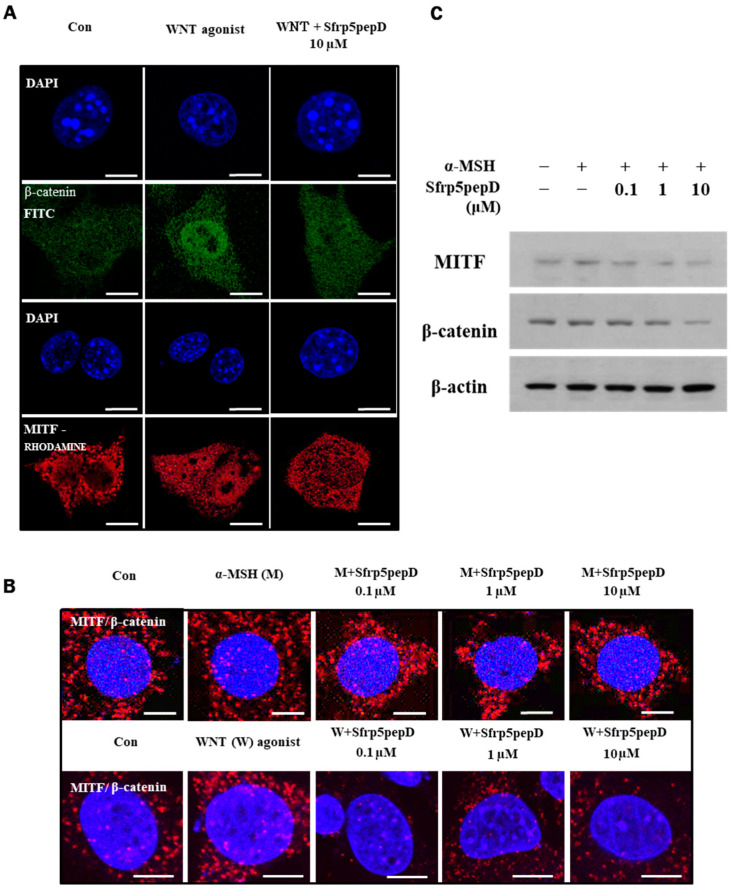
Nuclear localization of MITF and β-catenin are suppressed by SFRP5pepD. (**A**) B16F1 cells were treated with Wnt agonist or Sfrp5pepD (10 μM) for 3 h. After fixation, immunofluorescent staining was conducted to detect the nuclear-translocation of MITF and β-catenin. Cells were incubated with an anti-MITF or β-catenin mAb and then phalloidin-rhodamine-conjugated anti-rabbit or FITC-conjugated anti-mouse IgG antibody. (**B**) B16F1 cells were treated with α-MSH/Wnt agonist or Sfrp5 peptides (1 μM) for 12 h. After fixation, in situ PLA for MITF/β-catenin was performed with specific antibodies. Nuclei were stained with DAPI (blue). Red spots represent physical interaction between the two indicated molecules. Scale bar = 10 μm. (**C**) The protein expressions of the MITF and β-catenin were examined using Western blot analysis.

We investigated whether Sfrp5pepD reduces the nuclear localization of MITF and β-catenin by inhibiting Wnt signaling. Immunofluorescence analyses were conducted on B16 melanoma cells using antibodies to β-catenin (green) or MITF (red). As anticipated, Wnt agonist increased the nuclear localization of MITF and β-catenin, whereas Sfrp5pepD inhibited it (see Figure 2A).

Moreover, Sfrp5pepD dose-dependently suppressed the physical interaction between MITF and β-catenin (see Figure 2B). Subsequently, we investigated whether Sfrp5pepD downregulates the expression of MITF and β-catenin proteins using Western blot analysis. The protein expressions of MITF and β-catenin were reduced by Sfrp5pepD in a dose-dependent manner (see Figure 2C). These findings suggest that Sfrp5pepD inhibits the interaction between MITF and β-catenin by suppressing the activity of the Wnt/β-catenin signal. Other examples of peptides are rare, but a few exist. Disheveled PDZ peptides inhibit Wnt/β-catenin signaling in cells [41]. However, this is limited to research on cancer treatment.

### 3.3. Sfrp5pepD Inhibits Melanin Synthesis and the Expression of Melanogenic Enzymes in B16F1

MITF acts as a key transcription factor, promoting the increased expression of melanogenic enzymes like TYR, TRP-1, and DCT(TRP-2) [42]. Since Sfrp5pepD regulates MITF, measuring these related enzymes is crucial. During melanogenesis, α-MSH induction of MITF was mediated by a decrease in small heterodimer partner-interacting leucine zipper protein expression in B16F10 mouse melanoma cells [43]. For these reasons, it is important to confirm whether α-MSH-induced MITF inhibition, leading to the suppression of TYR, TRP-1, and TRP-2 activity, is indeed inhibited by the candidate substance.

**Figure 3:**
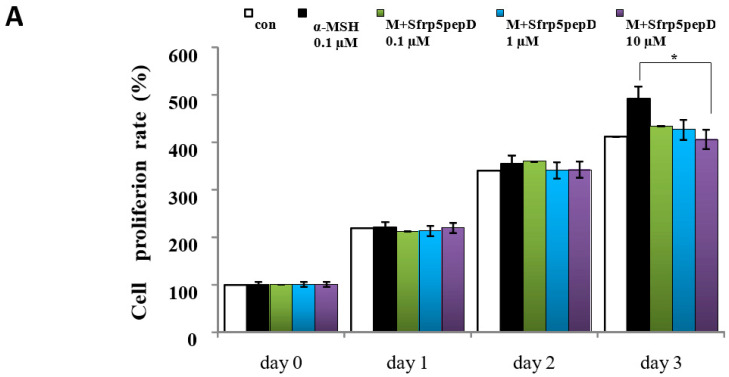

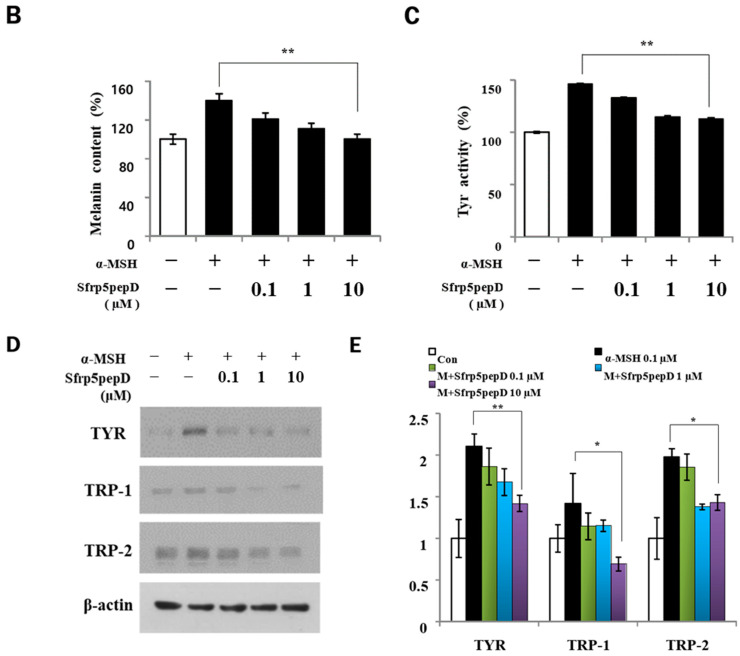
Sfrp5pepD inhibits melanin synthesis in B16F1 melanoma cells. (**A**) B16F1 cells were treated with 0.1, 1, 10 μM of Sfrp5pepD in conjunction with α-MSH (100 nM) for 3 days. The cellular growth rate of each sample was determined using the CCK-8. Significant differences were found when comparing with α-MSH-treated control: * *p* < 0.05. (**B**) The melanin content and (**C**) tyrosinase activity were determined by measuring the absorbance at 490 nm. B16 melanoma cells were treated with 0.1, 1, 10 μM of Sfrp5pepD in conjunction with 100 nM α-MSH for 48 h. The values indicate the mean of three independent experiments. Significant differences were found when comparing with α-MSH-treated control: ** *p* < 0.01. (**D**) The expression of TYR, TRP-1, TRP-2 mRNA was investigated via a real-time PCR analysis. The values indicate the mean of five independent experiments. (**E**) The protein expressions of the melanogenesis-associated enzymes, TYR, TRP-1, and TRP-2 were examined using Western blot analysis. Significant differences were found when comparing with α-MSH-treated control: * *p* < 0.05, ** *p* < 0.01.

To determine whether Sfrp5pepD affects cell proliferation, we performed CCK-8 assay on B16 melanoma cells treated with α-MSH or SFRP5pepD for 3 days (Figure 3A). Sfrp5pepD was found to inhibit the α-MSH-induced proliferation of B16F1 melanoma cells. We subsequently examined the melanin content and TYR activity to investigate how Sfrp5pepD affects melanogenesis (Figure 3B,C). Our findings indicated that α-MSH treatment increases the melanin contents in B16 melanoma cells, while Sfrp5pepD dose-dependently inhibits the α-MSH-induced melanin contents. The TYR activity using L-DOPA staining was also significantly reduced by SFRP5pepD.

In addition, we performed qRT-PCR and Western blot analysis on mRNA and the protein expression of melanogenic enzymes (Figure 3D,E) and found that Sfrp5pepD inhibits the expression of TYR, TRP-1, and TRP-2 protein. Consistent with the Western blot analysis, the quantitative real-time PCR analysis showed that α-MSH-induced expression of TYR, TRP-1, and TRP-2 mRNA was suppressed by the treatment with Sfrp5pepD in a dose-dependent manner. These data suggest that Sfrp5pepD has an inhibitory effect on melanogenesis in B16F1 melanoma cells. Inhibiting the Wnt signaling pathway leads to reduced melanin production. SFRP5 was overexpressed in the skin lesions of vitiligo patients. Indeed, SFRP5 inhibits melanin synthesis in melanocytes in vitiligo by suppressing the Wnt/β-catenin signaling pathway [30]. Sfrp5pepD also could inhibit the Wnt signaling pathway. These results are consistent with a series of findings related to Sfrp5pepD.

### 3.4. Sfrp5pepD Decreases Melanin Synthesis and the Expression of Melanogenic Enzymes in NHMs

**Figure 4:**
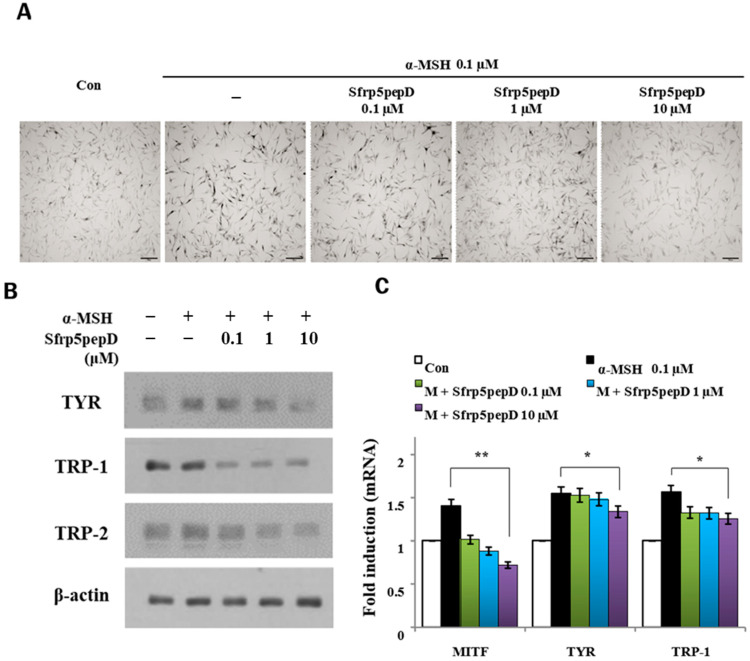
SFRP5pepD decreases melanogenesis in normal human melanocytes. (**A**) In situ tyrosinase activity was determined by the incubation of cells in L-DOPA, following 24-h treatment with 0.1, 1, and 10 μM of Sfrp5pepD. Each image was captured under identical conditions using bright field microscopy (bar = 100 µm). (**B**) Normal human melanocytes were stimulated with α-MSH and 0.1, 1, 10 μM Sfrp5pepD and incubated for 5 days. The expression levels of TYR, TRP-1, and TRP-2 protein were confirmed by Western blot at 5 days. (**C**) Normal human melanocytes were stimulated with α-MSH and 0.1, 1, 10 μM Sfrp5pepD and were incubated for 48 h. The ex-pression of TYR, TRP-1, and TRP-2 mRNA were investigated via a real-time PCR analysis. The values indicate the mean of three independent experiments. Significant differences were found when comparing with α-MSH-treated control: * *p* < 0.05, ** *p* < 0.01.

To further investigate the impact of Sfrp5pepD on melanin synthesis in normal primary human melanocytes (NHMs), we conducted an extended analysis. Initially, L-dopa staining was employed to assess the influence of Sfrp5pepD on tyrosinase activity within NHMs (Figure 4A). Our findings revealed that Sfrp5pepD effectively attenuated in situ tyrosinase activity while maintaining cell viability. Following this, we proceeded to evaluate the modulation of melanogenesis-associated gene expression at both protein and mRNA levels (Figure 4B,C). Notably, our results demonstrated a significant reduction in the expression levels of TYR, TRP-1, and TRP-2 genes upon treatment with Sfrp5pepD. To validate these findings, we performed RT-PCR analysis, corroborating the downregulatory effect observed in Western blot analysis. Collectively, these results underscore the inhibitory role of Sfrp5pepD in α-MSH-induced melanogenesis in NHMs, thereby broadening our understanding of its mechanism of action and therapeutic potential.

## 4. Discussion

Melanin is an essential presence in skin defense, but its removal is socially crucial not only in aesthetic aspects but also due to the increasing societal issue of depression caused by skin discoloration [44]. Numerous peptides are used in cosmetics related to wrinkle reduction and regeneration [33]. However, there are few studies on peptides that inhibit melanin. While there are studies regulating MC1R antagonists associated with a-MSH and utilizing sequences of active peptides resembling a portion of α-MSH sequences [45].

Some components that inhibit melanogenesis by controlling Wnt have been reported. One example of a Wnt inhibitor that can act as a melanin inhibitor is ICG-001. ICG-001 is a small molecule that specifically inhibits the interaction between β-catenin and CREB-binding protein (CBP), which is a crucial component of the Wnt signaling pathway [46]. This inhibition can lead to a decrease in melanogenesis [47]. Another example is DKK1. DKK1 is a naturally occurring protein that inhibits Wnt signaling by binding to LRP5/6 co-receptors, preventing the activation of the pathway [48]. By reducing Wnt signaling, DKK1 can also reduce melanin production [49]. XAV939 is another Wnt inhibitor that targets tankyrase enzymes, leading to the stabilization of Axin and promoting the degradation of β-catenin [50]. This suppression of β-catenin reduces Wnt pathway activity and subsequently melanin production. However, research controlling crucial signaling proteins such as Wnt during melanogenesis is very sparse.

SFRP5, a member of the SFRP family, has been linked to several immune-related disorders, including rheumatoid arthritis [51], psoriasis [52], and type 2 diabetes mellitus [53], which are conditions similar to vitiligo [13]. Additionally, activation of the Wnt signaling pathway is closely associated with repigmentation in vitiligo. SFRP5 also effectively suppresses the activity of the Wnt signaling pathway [28]; SFRP5 can be considered effective on its own as it actually inhibits melanin production [30].

While SFRP5 itself appears promising, it does have a significant drawback. SFRPs typically have a molecular weight ranging between 30 and 40 kD [31], whereas topical absorption ideally requires a size under 500 Da [32]. This means it needs to be under 500 Da. For this reason, a smaller core fragment was needed.

This study explores the inhibitory effects of a novel peptide, Sfrp5pepD, on the Wnt/β-catenin signaling pathway and its implications for melanogenesis. Our findings indicate that Sfrp5pepD, derived from SFRP5, retains the Wnt inhibitory mechanism and effectively reduces melanin production in both melanoma cells and normal human melanocytes.

The Wnt/β-catenin signaling pathway is crucial for melanin synthesis, and α-MSH is a very important inducer in this mechanism [16]. We created four Wnt antagonist peptides (A, B, C, D), and found that Sfrp5pepD exhibited the most potent Wnt antagonist ability. It was determined that the AQK sequence is key to its activity. AQK comes from a cysteine-rich domain. The cysteine-rich domain in SFRPs enables them to bind to Wnt proteins [23], which prevents Wnt from interacting with its receptors on the cell surface. This binding is crucial for regulating the Wnt signaling pathway, which plays a significant role in various developmental processes and diseases [54].

Since the cysteine-rich domain of SFRP5 includes a segment known as AQK, which is part of Sfrp5pepD, SFRP5 effectively suppresses the activity of the Wnt signaling pathway [28]. It can be confirmed that Sfrp5pepD, derived from SFRP5, also possesses the same Wnt inhibitory mechanism. SFRP5 inhibits melanin synthesis in melanocytes in vitiligo by suppressing the Wnt/β-catenin signaling pathway [30], and we expect that Sfrp5pepD will have the same effect.

The Wnt/β-catenin signaling stabilizes the interaction between MITF and β-catenin [37,38,39]. Wnt can indeed regulate MITF at the protein degradation level, irrespective of its transcription [40]. Our findings indicate that Sfrp5pepD disrupts the Wnt/β-catenin signaling pathway by reducing the nuclear localization and interaction between MITF and β-catenin. An inhibitor of the canonical Wnt/β-catenin signaling pathway is accountable for suppressing melanocyte pigmentation by downregulating β-catenin and MITF expression [13].

This interference results in a dose-dependent decrease in the protein levels of MITF and β-catenin, highlighting the crucial role of the pathway in regulating melanogenesis. For SFRP5, this mechanism has been confirmed; SFRP5 overexpression suppresses melanin synthesis by reducing MITF expression [30]. The decrease in melanin synthesis observed with Sfrp5pepD treatment further supports its potential as a Wnt pathway inhibitor. This aligns with the fact that Wnt pathway inhibitors can reduce melanin production. Wnt5a, a noncanonical Wnt molecule, has been identified as an inhibitor of melanin synthesis [55].

Recent studies have also clarified that the increased stability of newly synthesized β-catenin contributes to the stability of MITF, thus activating Wnt signaling [56]. This indicates that if Sfrp5pepD inhibits Wnt signaling, it may reduce the activity of both MITF and β-catenin.

The inhibitory impact of Sfrp5pepD on melanogenesis was apparent through the decreased activity of melanogenic enzymes, such as TYR, TRP-1, and DCT (TRP-2), which play vital roles in melanin synthesis [57]. MITF serves as a pivotal transcription factor, stimulating the heightened expression of melanogenic enzymes like TYR, TRP-1, and DCT [42]. The peptide’s ability to suppress α-MSH-induced proliferation and melanin content in B16F1 melanoma cells highlights its therapeutic potential. These findings align with previous research on SFRP5, which shows inhibition of melanin synthesis through the suppression of the Wnt/β-catenin signaling pathway [30]. Indeed, SFRP5 inhibits melanin synthesis in melanocytes in vitiligo by suppressing the Wnt/β-catenin signaling pathway [30]. Sfrp5pepD also could inhibit the Wnt signaling pathway. These results are consistent with a series of findings related to Sfrp5pepD. Other examples of peptides are rare, but a few exist. Disheveled PDZ peptides inhibit Wnt/β-catenin signaling in cells; however, this research is limited to cancer treatment, not melanogenesis [41]. Sfrp5pepD decreases melanogenesis in B16 melanoma cells. Correspondingly, recent research indicates that Sfrp5 levels are notably increased in B16 cells treated with human NSC-CM [13]. NSC-CM may disrupt various factors’ associations, leading to reduced melanin production [13]. Conversely, Sfrp5pepD not only inhibits the nuclear translocation of MITF and β-catenin but also reduces their protein expression levels.

Peptides for effective topical absorption ideally have a size under 500 Da [32]. Sfrp5pepD, with a molecular weight below this threshold, emerges as a promising candidate for transdermal delivery, overcoming limitations associated with larger SFRP5 proteins. Unlike SFRPs, which have a molecular weight ranging between 30 and 40 kD [31], Sfrp5pepD’s smaller size enables it to comply with the 500 Da rule for skin permeation.

Moreover, Sfrp5pepD provides the benefits of small peptides. They are generally more stable than proteins under thermal or chemical stress [58]. Additionally, they are less likely to trigger an immune response compared to larger proteins [59]. They can be synthesized more easily and affordably than large proteins [60]. Also, small peptides are swiftly cleared from the body, aiding in minimizing side effects and facilitating rapid-action scenarios [61].

However, a limitation of this study is the absence of clinical trials. Thus, further clinical trials related to formulations containing Sfrp5pepD are warranted, particularly for skin-related applications. This characteristic significantly enhances its potential as a key ingredient in cosmetics or pharmaceuticals targeting pigmentary disorders. Our study’s findings suggest that Sfrp5pepD could serve as a potent inhibitor of melanin production, paving the way for further exploration in clinical applications. This outcome also implies that this substance holds promise not only as a therapeutic agent but also as a vital topical ingredient in cosmetic formulations.

Future studies should focus on the long-term effects and safety of Sfrp5pepD in clinical settings. Additionally, exploring the peptide’s impact on other pathways involved in skin pigmentation could provide a comprehensive understanding of its mechanism. Investigating Sfrp5pepD’s potential synergistic effects with other known melanogenesis inhibitors may also offer insights into more effective treatments for hyperpigmentation and related disorders.

## 5. Conclusions

Sfrp5pepD, a small peptide derived from SFRP5, is a suppressor of melanin synthesis. The presented evidence supports its mechanism of action and paves the way for future investigations into its therapeutic applications.

Key Findings:

- Wnt/β-catenin Pathway Inhibition: Sfrp5pepD disrupts the Wnt/β-catenin signaling pathway, a crucial regulator of melanogenesis, by hindering the interaction between Axin-1 and β-catenin.
- Reduced MITF/β-catenin Interaction: Sfrp5pepD treatment suppresses the nuclear translocation and physical interaction of MITF and β-catenin, essential factors in regulating melanin production.
- Melanin Synthesis Inhibition: Sfrp5pepD effectively inhibits melanin production and tyrosinase activity in B16F1 melanoma cells and normal human melanocytes (NHMs) stimulated by α-MSH.
- Melanogenic Enzyme Downregulation: Sfrp5pepD treatment leads to a dose-dependent decrease in the expression of key melanogenic enzymes, including TYR, TRP-1, and TRP-2.

Mechanism of Action: The results suggest that Sfrp5pepD targets the Wnt/β-catenin signaling pathway. By inhibiting this pathway, Sfrp5pepD disrupts the interaction between MITF and β-catenin, thereby downregulating the expression of melanogenic enzymes and ultimately reducing melanin synthesis.

Significance: This study highlights the potential of Sfrp5pepD as a promising therapeutic agent or cosmetic active ingredient for hyperpigmentation disorders. Its ability to target a specific pathway involved in melanogenesis offers a promising alternative to existing treatments. Additionally, the smaller size of Sfrp5pepD compared to SFRP5 enhances its potential for transdermal delivery in topical applications.

Future Directions:

- Clinical Trials: Clinical trials are crucial to determine the effectiveness and potential side effects of Sfrp5pepD in human subjects.
- Delivery Methods: Developing efficient delivery systems for topical application of Sfrp5pepD is essential for maximizing its therapeutic potential.

## Data Availability

The data that support the findings of this study are available from the corresponding author upon reasonable request.
